# Finite Element Modelling of Corrosion-Damaged RC Beams Strengthened Using the UHPC Layers

**DOI:** 10.3390/ma15217606

**Published:** 2022-10-29

**Authors:** Mohammed A. Al-Huri, Mohammed A. Al-Osta, Shamsad Ahmad

**Affiliations:** 1 Department of Civil & Environmental Engineering, King Fahd University of Petroleum and Minerals, Dhahran 31261, Saudi Arabia; 2 Interdisciplinary Research Center for Construction and Building Materials, King Fahd University of Petroleum and Minerals, Dhahran 31261, Saudi Arabia

**Keywords:** reinforced concrete (RC) beams, reinforcement corrosion, strengthening, ultra-high-performance concrete (UHPC), finite element modelling (FEM), parametric study

## Abstract

This paper describes a study on finite element modeling (FEM) carried out on the ABAQUS platform for the prediction of flexural strength of corrosion-damaged reinforced concrete (RC) beams strengthened using layers of ultra-high-performance concrete (UHPC). Considering different combinations of the degree of reinforcement corrosion and thickness and configuration of UHPC layers, a total of twenty-two corroded, un-strengthened, and strengthened RC beam specimens were tested to record their flexural behavior. Following the flexural testing, the FEM was carried out considering the degradation in the diameter and the yielding strength of the corroded reinforcing bars. The cohesive surface bonding approach was used to simulate the interfacial bond stress slip between the corroded bars and surrounding concrete. The results of the FEM were validated using the experimental test results of the respective beam specimens. The FEM results (including crack pattern, flexural strength, stiffness, and linear and nonlinear behavior of the strengthened RC beams) were found to be in close agreement with the corresponding experimental test results. This indicates that the proposed FEMs can capture the flexural behavior of the corroded RC beams strengthened using layers of UHPC with high accuracy. Furthermore, a parametric study was carried out using the validated FEMs to investigate the effects of varying the compressive strength and thickness of UHPC layers on the flexural strength of the corroded strengthened RC beams.

## 1. Introduction

Reinforcement corrosion in concrete structures is one of the main reasons behind the loss of load-carrying capacities sabotaging the service life of reinforced concrete (RC) elements. Reinforcement corrosion produces a variety of issues, including cracking and spalling of the concrete cover, loss of rebar diameter and strength, and loss of bond strength between rebars and surrounding concrete, which degrades structural integrity and serviceability, resulting in sudden collapse [1,2,3]. The loss of load-carrying capacities of the corroded RC beams is mainly due to the loss of cross-sectional area and reduction of the mechanical properties of the corroded steel bars, such as ductility, yield, and ultimate strength, leading to premature failure [4,5,6,7]. Furthermore, the bond between corroded steel bars and surrounding concrete is significantly affected by reinforcement corrosion that leads to cracking and spalling of the concrete cover of RC elements [8,9,10]. Prediction of the residual load-bearing capacity of the RC members damaged by reinforcement corrosion is crucial for existing structures for employing suitable repair and strengthening to augment the service life of damaged RC members. Therefore, developing efficient, safe, and cost-effective materials and methods for repairing and strengthening is of high interest.

A review of several studies pertaining to the corrosion of RC beams and various strengthening materials and methods is reported in the literature. Research works in the past have demonstrated the efficacy of fiber-reinforced polymers (FRP) as a trustworthy material for the strengthening of RC buildings [11,12,13]. Cement mortars reinforced with carbon, glass, or polyparaphenylene benzobisoxazole (PBO) fabrics, known as a fabric-reinforced cementitious matrix (FRCM) or textile-reinforced mortar (TRM), have lately been introduced as viable, sustainable, and durable alternatives to FRP composites. However, ultra-high-performance concrete (UHPC), an innovative cement-based composite material, has evolved recently [14]. Because of its dense homogeneous microstructure, UHPC has extremely high strength, modulus of elasticity, ductility, and durability [15,16,17]. The development of an ideal mixture for mixing and casting UHPC utilizing locally accessible resources in Saudi Arabia was reported [18]. The researchers have recently explored the utilization of UHPC as an alternate material for strengthening RC members. The structural behavior of using UHPC for strengthening RC beams and slabs was examined [19,20]. The flexural behavior of damaged RC beams strengthened by the toughness-improved ultra-high performance concrete (UHPC) layer was investigated [21]. Al-Osta [22] investigated the shear behavior of RC beams retrofitted with precast panels of UHPC using an appropriate epoxy adhesive. The strengthened beams significantly improved the load-bearing capacity, stiffness, and failure mode. The effect of using layers of UHPC on the shear behavior of RC beams was examined by Bahraq et al. [23]. Results of the strengthened beams showed the efficiency of retrofitting the RC beams using layers of UHPC which improved the shear capacity of the beam and shifted the failure mode from brittle to ductile. A study was conducted by Lampropoulos et al. [24] to examine the efficiency of using UHPC to strengthen conventional RC beams. The flexural capacity of UHPC-normal concrete composite beam without any shear stirrups was studied by Hussein and Amleh [25]. The results showed that both the flexural and shear behavior of the composite beam system improved.

The numerical analysis using FEM makes it possible to anticipate the exact response of the same structural elements with alternative UHPC configurations and thicknesses without conducting additional experimental testing. Modeling the RC beams with corroded longitudinal reinforcing bars requires an appropriate approach for the degradation of bond-slip behavior. Several researchers have adopted finite element modelling to evaluate the structural behaviour of strengthened RC beams, including predicting failure loads and cracking patterns. However, a big challenge in numerical simulation is the modelling of concrete cracking. Bažant and Oh [26] and Tao and Chen [27] adopted the crack band theory, in which the width of the concrete crack opening is related to the crack band width and crack strain, to eliminate the mesh sensitivity problems in FE simulation. Lampropoulos et al. [24] and Sadouki et al. [28] examined the UHPC-strengthened RC beams using the smeared crack technique. Good agreement between the experimental and numerical modeling results of the load-deflection behavior was observed. However, it was reported by Al-Osta et al. [29] that the concrete damage probabilistic model is the most widely used for simulating cracking in concrete members. Al-Osta et al. [29] and Murthy et al. [30] used the concrete damage plasticity theory to develop a finite element model that simulates the non-linear behavior of UHPC-strengthened RC beams in tension and compression. Results showed that the suggested model could predict the load-deflection response and crack patterns in agreement with the respective experimental results. Several researchers initially employed the concrete damage plasticity model (CDPM) to simulate the behavior of conventional concrete [31]. However, it was found in the literature that the CDPM can be used to model both conventional concrete and UHPC material in ABAQUS with reasonable accuracy [16,23,29].

Three alternative methodologies to simulate the rebars-concrete bond for modeling steel reinforcing bars in the RC beams using finite element (FE) analysis have been reported in the literature, as described below:

- The first one involves using a spring element to transfer tension between rebar and concrete. This approach is useful in 2D modeling, where only two nodes represent steel bars as truss elements. The nonlinearity of spring elements can be specified by inserting the experimental load against the displacement relationship. Li et al. [32] employed the 1D interface element, a translator in ABAQUS, to conduct a numerical analysis to study the behavior of corroded RC seawalls representing the bond by the element having two nodes linking the concrete and the steel reinforcement bars. Xiaoming and Hongqiang [33] utilized the spring interface parts to simulate the bond behavior between corroded bars and concrete. However, the results were not validated against experimental test results. Ou and Nguyen [34], Hanjari et al. [35], Kallias and Imran Rafiq [36], and Coronelli and Gambarova [37] used interface components to model the rebars-concrete bond behavior in 2D finite element modeling for corroded bars in RC members under flexure. Other researchers, for example, Kallias and Imran Rafiq [36], Val and Chernin [38], and Murcia-Delso and Shing [39], used four-noded interface elements to simulate the bond strength in ABAQUS.
- The second approach to simulate the loss of bond strength between steel bars and concrete consists of modifying the properties of concrete and steel. To demonstrate the bond interaction between steel bars and concrete, Ziari and Kianoush [40] adjusted the characteristics of concrete in contact with the reinforcing bars in a small region, known as the bond zone, in which the tensile strength and fracture energy of concrete elements were lowered. Dehestani and Mousavi [41] investigated the bond interaction by adding the equivalent strain due to the bond slip to the strain of the steel bars.
- The third approach simulates the connection as an interaction between three-dimensional surfaces of concrete and steel, as proposed by Amleh and Ghosh [42] for finite element modeling of corroded and non-corroded RC members using the results of the pullout tests. This approach can be applied to 3D concrete and steel models in ABAQUS utilizing the mechanical contact property for simulating the tangential and normal behavior of concrete and steel bars contacting surfaces. Biondini and Vergani [43] and Almassri et al. [44] performed 3D FEM on corroded RC beams without considering the loss of bond. German and Pamin [45] performed 2D and 3D FEM of corroded steel bars with corrosion product (rust) modeled as an interface element (COH2D4 elements) in the nonlinear FEM software using ABAQUS.

Although some previous studies on the application of UHPC in strengthening undamaged RC beams are reported in the literature, as briefly described above, there is a lack of data on the performance of UHPC strengthening of corroded RC beams. Furthermore, there is not enough information available on the numerical modelling of corroded RC beams after strengthening with different configurations and thicknesses of UHPC layers. Therefore, the work presented in this paper was aimed mainly at bridging this gap. The present study consisted of experimental investigation (to generate the data pertaining to the flexural behavior of the corroded strengthened RC beams) and finite element modelling of the respective RC beams using ABAQUS to capture their structural behavior. The experimental investigation included strengthening the corroded RC beams with three different UHPC thicknesses and two various configurations. The FE simulation of the corroded strengthened and un-strengthened beam specimens was carried out using the fundamental knowledge of structural behavior and properties of the RC beams and UHPC strengthening layers. The commercial finite element analysis (FEA) software, ABAQUS/CAE 2017, was used to model the RC beam specimens using its sophisticated numerical capabilities and extensive choice of concrete material models. The load versus mid-span deflection curves were obtained from the results of numerical modelling and validated against the experimental test results. Furthermore, the FEM was used for parametric study to evaluate the effect of varying the compressive strength and thicknesses of the UHPC layers on the ultimate load-carrying capacities of the corroded strengthened RC beams.

## 2. Experimental Program

### 2.1. RC Beam Specimens

The test parameters included the degree of reinforcement corrosion and configuration and thicknesses of UHPC layers applied on the bottom and side surfaces of the RC beams. The details of the test specimens are shown in Table 1. One RC beam specimen, neither corroded nor strengthened (*UU*), was used as the control specimen, while the remaining twenty-one RC beam specimens were divided into three groups (*A*, *B*, and *C*) and exposed to accelerated reinforcement corrosion using the impressed current technique to obtain theoretical mass losses of 10, 20, and 30%, respectively, in their longitudinal tensile steel bars. The RC beam specimens were connected in series to the accelerated corrosion setup, and a constant current density of 200 µA/cm^2^ was applied for the entire period of the corrosion process as recommended by El Maaddawy and Soudki [46]. After inducing reinforcement corrosion, one beam specimen from each group was tested without strengthening (*CUA*, *CUB*, and *CUC*). The remaining corroded RC beams were strengthened either from one side (donated by the letter *R*) or from three sides (donated by the letter *S*). Three different thicknesses of UHPC layers were considered for both one-sided and three-sided strengthening configurations: 20, 40, and 60 mm for one-sided and 20, 30, and 40 mm for three-sided.

**Figure 1:**
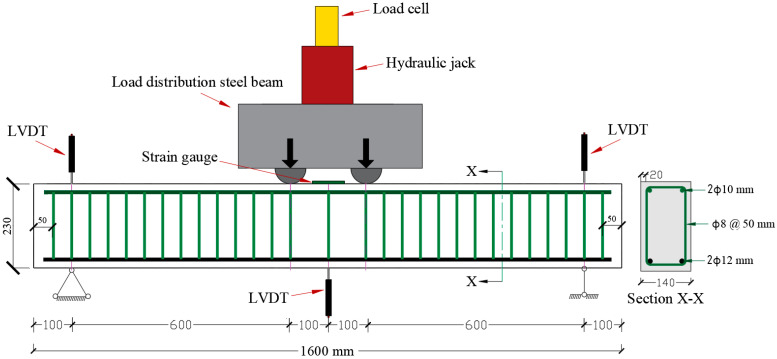
Geometry, reinforcement details, and test setup configuration of the RC beam specimens (unit: mm).

**Figure 2:**
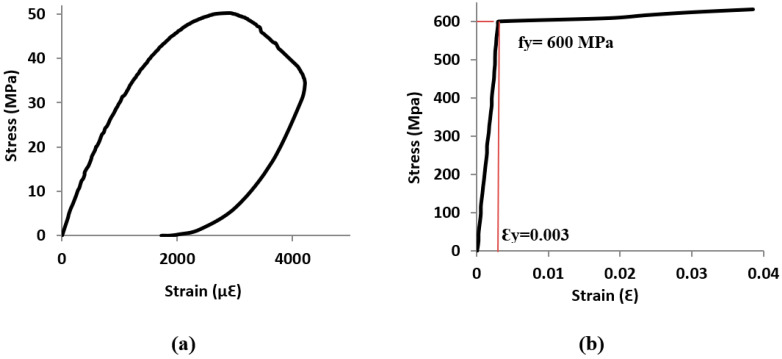
Stress-strain curves of (**a**) normal concrete (**b**) tensile steel bars.

The geometry and reinforcement details of the RC beam specimens and the flexure test setup are shown in Figure 1. As can be seen in Figure 1, the RC beam specimens were 1600 mm long with a rectangular cross-section of 140 mm wide and 230 mm deep. The beam specimens were reinforced at the bottom with 2 Φ 12 mm deformed bars. On the top side, 2 Φ 10 mm deformed bars were used as a hanger to support shear stirrups. To avoid shear failure of the beams, Φ 8 mm stirrups were placed at a spacing of 50 mm along the entire length of the beams. The RC beams were loaded to ensure a mid-span deflection at a constant rate of 0.5 mm/min. The nominal yield strengths of the bottom and top reinforcing bars were 600 and 580 MPa, respectively, with a standard deviation of 22 and 19 MPa, respectively. Stirrups had a yield strength of 550 MPa with a standard deviation of 24 MPa. The elastic modulus of all the steel bars was found to be 200 GPa. The tensile tests of the reinforcing steel bars were conducted at a loading rate of 150 MPa/min as per ASTM E8/E8M [47]. The actual mass losses of the tensile rebars in the RC beams were used to determine the degradation in the yield strength and elastic modulus of the corroded steel bars that were utilized in the FE simulation. The actual mass losses of the corroded rebars were measured in accordance with ASTM G1 standards [48], and the results are presented in Table 1. Normal concrete used to cast the RC beam specimens was composed of ASTM C150 [49] Type-I cement, natural dune sand, crushed limestone, sweet water, and superplasticizer. The compressive strength and elastic modulus of normal concrete were determined as per ASTM C39 standards [50] at a loading rate of 14 MPa/min. The compressive strength and elastic modulus of the normal concrete were determined as 50 and 33,000 MPa with a standard deviation of 3.8 and 1100 MPa, respectively. In addition, the splitting tensile test of normal concrete was conducted in accordance with ASTM C496 standards [51] at a loading rate of 1.05 MPa/min, and the splitting tensile strength was found to be 3.3 MPa with a standard deviation of 0.28 MPa. The typical stress-strain curves of normal concrete and tensile steel bars used in the FEM are shown in Figure 2.

### 2.2. UHPC Strengthening: Properties, Configurations, and Bond Strength

**Figure 3:**
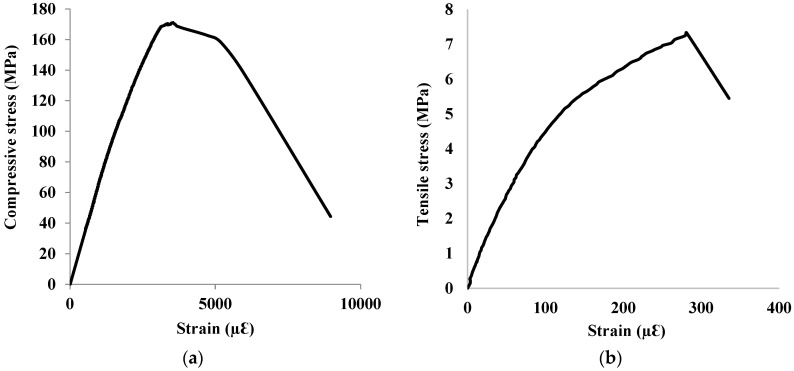
Plot of stress-strain curves for UHPC in (**a**) compression (**b**) direct tension.

The compressive strength and elastic modulus of the UHPC mixture used for strengthening the corroded RC beam specimens were measured as 170 MPa and 46 GPa with a standard deviation of 7.5 MPa and 1.1 GPa, respectively. UHPC had a direct tensile strength of 7.3 MPa with a standard deviation of 0.46 MPa. Loading rates of 18 and 1.5 MPa/min were used to determine the compressive and tensile strength of UHPC, respectively. The stress-strain curves for UHPC tested in compression and direct tension, as shown in Figure 3, were utilized in the FEM of the UHPC strengthening layer in ABAQUS.

**Figure 4:**
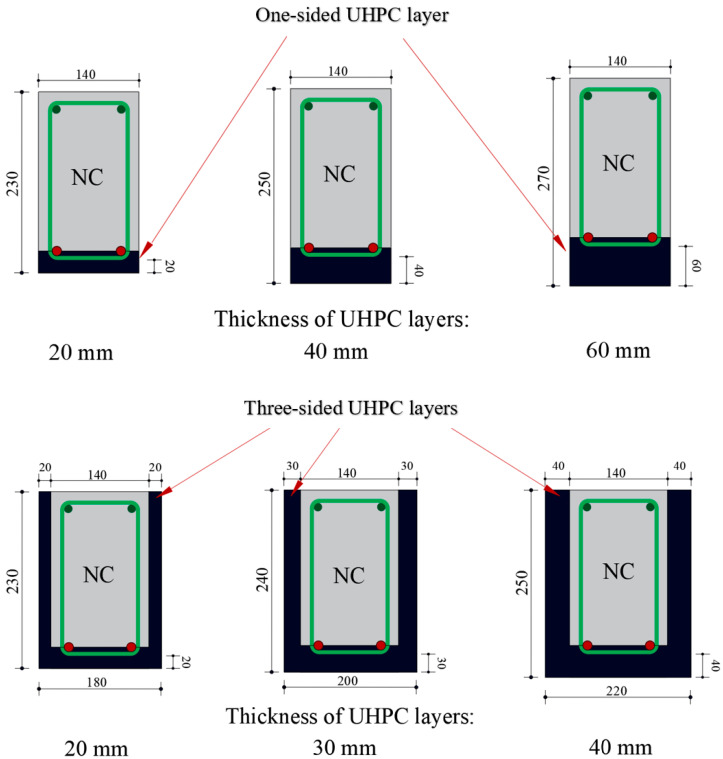
Configurations and thicknesses of UHPC strengthening layers.

The UHPC strengthening using different thicknesses and configurations applied on the corroded RC beams is shown in Figure 4. To improve the bond strength between UHPC layers and original RC beams, sandblasting was performed on the surfaces of the corroded beam specimens. To simulate the bond between UHPC and normal concrete in ABAQUS, the bond strength of UHPC was measured experimentally using the split tensile test and slant shear test as described in the literature [52]. The test specimens were prepared similarly to that used in the experimental testing of the strengthened RC beam specimens. The average tensile bond strength and shear bonding strength were measured as 4.5 MPa and 18 MPa, respectively, with a standard deviation of 0.5 MPa and 1.1 MPa, respectively. Both test results showed higher values than the minimum values recommended by the ACI 546 R-14 [53] for an adequate bond between UHPC and concrete. In addition, no debonding was observed during the experiments for either of the two UHPC configurations. Therefore, the assumption of a perfect bond between UHPC and normal concrete can be valid during the FE simulation of strengthened RC beams.

## 3. Finite Element Simulation

Three-dimensional FE models were developed to simulate the nonlinear flexural response of the corroded strengthened and un-strengthened RC beam specimens using the nonlinear software package ABAQUS. The numerical simulation was carried out to confirm the adequacy of the experimental results for highlighting the flexural behavior of corroded strengthened RC beams that included the depiction of load versus deflection plots, ultimate strength, and crack patterns. The entire RC beam was modelled in the software, and the concrete damage plasticity model (CDPM) was used for modelling both normal concrete and UHPC. The effect of mass loss of the reinforcing bars due to reinforcement corrosion was taken care of by considering reductions in the yield strength and modulus of elasticity of the corroded steel bars. The reductions in the yield strength and modulus of elasticity of the corroded steel bars were calculated using the actual mass losses obtained from the gravimetric analysis and given in Table 1. The mechanical properties of the normal concrete and UHPC reported earlier were used as input data in the 3D modelling. The constitutive laws of different materials, bond simulation, element types, model constraints, and boundary conditions that are used in developing the FE models are presented in the following sections.

### 3.1. Concrete Damage Plasticity Model (CDPM)

The concrete damage plasticity model (CDPM), a powerful tool for predicting concrete behavior under both static and dynamic loading conditions, was adopted in the present work. The CDPM was utilized in the FEM because it is more stable for numerical computations, particularly in failure that shows a softening bias. The CDPM was initially developed by Lubliner et al. [54] and improved by Lee and Fenves [55]. It simulates the constitutive behavior of concrete by offering the scalar damage variables in compression and tension. It works in two different mechanisms; compressive crushing and tensile cracking concrete. In the tension mechanism, the concrete employs a multi-axial damage elasticity model, while in the compression mechanism, it employs a multi-axial plasticity model with non-associated flow and isotropic scalar hardening. Equivalent plastic strains in tension and compression are two hardening variables that characterize damage states in compression and tension separately. The increment values of the variables of the hardening mechanism produce compression crushing and tension cracking; these variables control the development of the yield surfaces and degradation of the elastic stiffnesses.

The CDPM variables in compression and tension are denoted by $d_{c}$ and $d_{t}$, respectively. The values of $d_{c}$ and $d_{t}$ range from zero (for undamaged material) to one (for completely damaged) [56]. Two failure mechanisms can be described by utilizing the CDPM approach in FEM: compressive crushing and tensile cracking. The yield surface in the deviatory plane is controlled by two hardening variables $\epsilon_{c}^{pl,h}$ and $\epsilon_{t}^{pl,h}$, which are related to the failure mechanisms under compression and tension, respectively. The $d_{c}$ and $d_{t}$ were calculated based on the equations developed by Birtel and Mark [57] as follows:

(1) $$d_{c}=1-\frac{\sigma_{c}\;\;E_{c}^{-1}}{\epsilon_{c}^{pl,h}\left(\frac{1}{b_{c}}-1\right)+\sigma_{c}\;\;E_{c}^{-1}}$$

(2) $$d_{t}=1-\frac{\sigma_{t}\;\;E_{c}^{-1}}{\epsilon_{t}^{pl,h}\left(\frac{1}{b_{t}}-1\right)+\sigma_{t}\;E_{c}^{-1}}$$

where: $\sigma_{c}$ and $\sigma_{t}$ are compressive and tensile stresses of concrete, $E_{c}$ is the elastic modulus of concrete, $\epsilon_{c}^{pl,h}$ and $\epsilon_{t}^{pl,h}$ are the plastic strains corresponding to compressive and tensile strengths of concrete, and $b_{c}$ and $b_{t}$ are constant parameters ranging between zero and one.

To model the concrete behavior using the CDPM, the following input parameters were defined in ABAQUS:

- The dilation angle is defined in the p-q plane at high confining pressure (*Ψ*).
- The parameter of eccentricity (*ϵ*).
- The initial ratio of equi-biaxial compressive yielding stress to the initial uniaxial compressive yielding stress ($\frac{\sigma_{b0}}{\sigma_{c0}})$.
- The ratio of the tensile to the compressive meridian defines the shape of the yield surface in the deviatory plane (K).
- Parameter of viscosity.

Table 2 shows the input parameter values for normal concrete and UHPC used in the FEM to define the CDPM following the information available in the literature for modeling the nonlinear behavior of both normal concrete and UHPC [29,58]. The nonlinear behavior of normal concrete and UHPC material was simulated in ABAQUS by directly inputting the required CDPM parameters and the nonlinear behavior of the materials (results obtained from experimental tests) into the selected model [59]. Different models were developed in this study considering different values of dilation angle (ranging between 15 and 40°) for normal concrete and UHPC. The results showed insignificant variation in the load-deflection curves obtained using the developed FEMs. A dilation angle of 36° was adopted in this study for both normal concrete and UHPC based on several research works reported in the literature [29,60].

### 3.2. Bond Simulation of Corroded Bars Using Cohesive Surface Bonding Approach

Unlike the case of finite element modeling of RC beams without corrosion, the assumption of the perfect bond cannot be used for modeling the RC beams with corroded steel bars since the bond of the corroded bars is significantly affected by the reinforcement corrosion. Therefore, the corroded bars were simulated using 3D surface interaction modeling methods available in ABAQUS to simulate the bond behavior between the corroded bars and surrounding concrete in a better way. In order to simulate the bonded surfaces between corroded steel reinforcement bars and surrounding concrete using a cohesive surface bonding approach, the surface-based cohesive behavior can be used to express the linear elastic relationship between two different surfaces due to its effectiveness and convenience [61]. The initial linear elastic behavior and the post-elastic behavior, characterized by the initiation and development of bond degradation, are the two components of the traction-separation model in ABAQUS. The elastic behavior is represented by an elastic constitutive matrix, which connects shear and normal stresses to shear and normal separations at the interface [56]. The constitutive equation for the initial elastic part is defined using Equation (3).

(3) $$t=\left(\begin{array}{c} t_{n}\\ t_{s}\\ t_{t} \end{array}\right)=\left(\begin{array}{c} K_{nn}\\ 0\\ 0 \end{array}\;\;\;\;\begin{array}{c} 0\\ K_{ss}\\ 0 \end{array}\;\;\;\;\begin{array}{c} 0\\ 0\\ K_{tt} \end{array}\right)\left(\begin{array}{c} \delta_{n}\\ \delta_{s}\\ \delta_{t} \end{array}\right)=K\delta \;\;\;\;\;\;\;$$

where: $K_{nn}$, $K_{ss}$, $K_{tt}$ are the stiffness elements [force/length^2^/length] that define the contact in normal, shear, and tangential directions, respectively. The relationship between the bond stress and the slip of corroded steel bars can be used to simulate the post-elastic behavior, which is indicated by the initiation and evolution of bond deterioration. The bond damage criterion is activated when the associated stresses exceed a maximum allowable value, which can be used to approximate this relationship. To simulate the bond behavior between corroded steel bars and concrete in ABAQUS using the cohesive surface bonding approach, parameters that define the interaction behavior of the 3D surfaces were determined to capture the exact response of the bond-slip relationship between the two surfaces. Several models were reported in the literature to model the concrete-steel interaction for the RC elements. The following equations can be used to estimate the shear and normal stiffness elements that were used in ABAQUS.

(4) $$k_{ss}=k_{tt}=\frac{\tau_{\max}}{S_{\max}}$$

(5) $$k_{nn}=100\;k_{tt}$$

where: $k_{nn}$, $k_{ss}$, $k_{tt}$, are the stiffness elements in the normal, shear, and tangential directions, respectively. $\tau_{\max}$ is the bond strength of corroded steel bars. $S_{\max}$ is the slip corresponding to the maximum bond stress. The maximum bond strength $\tau_{\max}$ of the corroded and un-corroded rebars in the RC, elements can be determined using Equation (6), proposed by El Maaddawy et al. [62]. Equation (6) contains two major terms: the first term is the influence of concrete, and the second term is the influence of shear stirrups.

(6) $$\tau_{\max}=R\;\left(0.55+0.24\;\frac{C_{c}}{d_{b}}\right)\;\sqrt{{\mathrm{fc}}^{\prime }}+0.191\;\frac{A_{t}{\;f}_{yt}}{S_{S}d_{b}}\;$$

where: R is the reduction factor for bond loss. $c_{c}$ is the smaller of one-half of the clear spacing between rebars and clear concrete cover. $d_{b}$ is the diameter of anchored steel bars. $S_{s}$ is the spacing between shear stirrups. $A_{t}$ is a cross-sectional area of stirrups within $S_{s}$. $f_{yt}$ is the yielding stress of stirrups.

The slip at maximum bond stress ($S_{\max}$) can be calculated using Equation (7), proposed by Kallias and Rafiq [36]:

(7) $$S_{\max}=0.15C_{0}e^{\frac{10}{3}\ln\left(\frac{\tau_{\max}}{\tau_{1}}\right)}+S_{0}\ln\left(\frac{\tau_{1}}{\tau_{max}}\right)\;$$

where: $C_{0}$ is rib spacing of the reinforcing bars. $\tau_{max}$ is the maximum bond strength. $\tau_{1}$ is the bond strength in well-confined concrete equal to $2.57\sqrt{{\mathrm{fc}}^{\prime }}$. $S_{0}$ is equal to 0.15 and 0.4 mm for plain and steel confined concrete, respectively.

To simulate the bond degradation of the corroded bars based on the cohesive surface bonding approach, a reduction in the $\tau_{max}$ is made using a reduction factor, R (as in Equation (6)) [61,62]. The reduction factor, R, can be estimated using Equation (8), proposed by Maaddawy et al. [62].

(8) $$R=A_{1}+A_{2}X_{p}\;\;\;\;<1$$

where: $A_{1}$ and $A_{2}$ are constant values depending on the corrosion current density used during the accelerated corrosion process, as proposed by Maaddawy et al. [62]. $X_{p}$ is the actual percentage mass loss of the reinforcing steel bars.

The Equations (4)–(8) were used to calculate the values of stiffnesses, $\tau_{max}$, and $S_{\max}$ used in the bond simulation of corroded steel bars in the FEMs, as shown in Table 3.

### 3.3. Elements Type and Meshing

**Figure 5:**
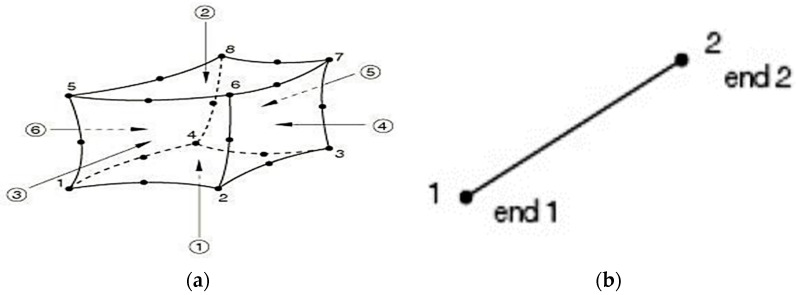
Finite element modeling meshing type. (**a**) C3D8R element, (**b**) T3D2 element.

**Figure 6:**
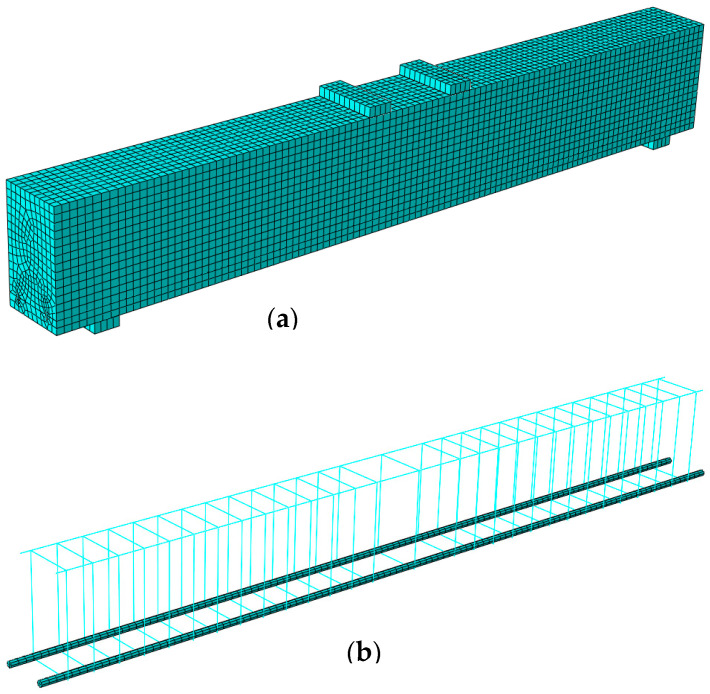
Discretization of the concrete beam (**a**) concrete elements (**b**) steel bars and stirrups.

**Figure 7:**
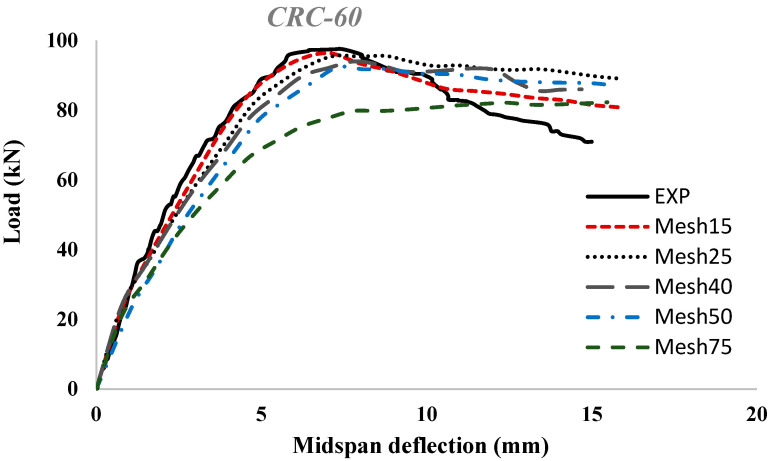
Load versus mid-span deflection curves for CRC-60 using different mesh sizes.

In order to model normal concrete, UHPC, and main steel bars, 3D stress eight-nodded linear brick elements (C3D8R) were used (solid continuum). This element type is suitable for linear as well as composite nonlinear analyses involving contact, deformations, and plasticity. Furthermore, this element was recommended in the ABAQUS Analysis user’s manual to avoid the shear-locking effect. In addition, steel plates were modeled at the loading points and supports to avoid the stress concentration of the eccentric loads and ensure uniform load distribution to the surface of the steel plates. The same FEM elements (C3D8R) were used to model the steel plates, and a perfect bonding behavior was assumed between the steel plated and normal concrete and/or UHPC. The support plates were restrained from movement in the transverse and vertical directions. In addition, two-nodded linear 3D truss elements (T3D2) were used to model the top steel reinforcement bars and stirrups. Typically, this structural element is long and slender and only transmits the axial loads. It allows the cross-section area of the truss elements to be defined, and the steel reinforcement can be shaped accordingly. Figure 5a,b shows the finite element modeling meshing type used in the numerical simulation. Figure 6a,b shows the discretized concrete beam elements and the discretized elements for the tensile steel bars and stirrups that were used in the finite element modeling in the present study. The element sizes of normal concrete and UHPC layers were approximately 15 × 15 mm. However, based on the mesh sensitivity study, increasing the element size to 25 × 25 mm significantly influenced the FEM results. Figure 7 shows the results of numerical modeling of the strengthened RC beam (*CRC-60*) using different mesh sizes ranging from 15 to 75 mm. The mesh size of 15 × 15 mm showed the best results in terms of stiffness, ultimate load, and overall flexural behavior, as evidenced by the close matching of the load-deflection curves obtained using FEM, considering a mesh size of 15 mm, and the experimental test results.

### 3.4. Finite Element Modeling of Corroded Steel Bars

As mentioned earlier, the degradation of the corroded steel bars due to reinforcement corrosion can be represented by a reduction in the yield strength and modulus of elasticity of the corroded bars, including the reduction in the cross-sectional area of the corroded steel bars. This reduction approach allows the utilization of the same rebar 3D element for various degrees of reinforcement corrosion. The reduction in the yield stress and elastic modulus of the corroded steel bars due to reinforcement corrosion was reported by Al-Osta et al. [61]. The actual mass loss values for each RC beam specimen were utilized in the material properties of the corroded steel bars in the FEM. The reduced yielding strength ($f_{yc}$) and elastic modulus ($E_{sc}$) of the corroded steel bars can be estimated based on the actual amount of corrosion mass loss using the following equations proposed by Al-Osta et al. [61].

(9) $$f_{yc}=\left(1-0.011X_{p}\right)\;f_{y}$$

(10) $$E_{sc}=\left(1-0.007X_{p}\right)\;E_{s}$$

where: $f_{y}$ is the un-corroded steel rebar’s yield stress, $E_{s}$ is the un-corroded steel rebar’s elastic modulus, and $X_{p}$ is the actual mass loss (%) of the rebars obtained from the gravimetric analysis.

### 3.5. Model Constraints

In order to model the RC beam specimens in ABAQUS, a number of constraints were applied. These constraints determined the interactions between various parts of the FEM and the analysis of the degree of freedom (DOF) between different regions of the model. The following subsections illustrate the constraints used in the modeling in the present study.

#### 3.5.1. Tie Constraint

The bond between normal concrete and UHPC was considered perfect since no debonding was observed during the flexural testing. The usage of tie constraint enabled the combination of the normal concrete beam and UHPC jacketing. However, the meshes created on the normal concrete regions and the UHPC layers differed. As the UHPC surfaces are harder, they were considered the master surfaces, whereas the normal concrete beam surfaces were slave surfaces. Because of the tie constraint, the translation, rotation, and other degrees of freedom were considered equal for a normal concrete-UHPC composite. In addition, the bond between steel plates and concrete surfaces was modeled using tie-bond constraint.

#### 3.5.2. Embedded Region Constraint

In order to model the interaction between top reinforcement steel bars (hangers) and stirrups with the concrete beams, the constraint of embedded regions was used, assuming a perfect bond. Considering the embedded region constraint, the stirrups and top steel bars are referred to as embedded regions in the concrete (i.e., host regions). Constraints were imposed based on the geometric relations between embedded and host elements. The translational DOF of the embedded nodes were controlled by the interposed values of their host elements corresponding to the DOF. In order to model the embedded elements in host elements (i.e., top bars and stirrups embedded in normal concrete), a truss-in-solid model has been used.

### 3.6. Analysis of FEM

The explicit dynamic analysis was implemented in the numerical modeling. This type of analysis was chosen because it can effectively address static issues, such as complex contact problems in quasi-static process modeling [63]. This is because of the fact that explicit dynamic analysis allows the solver to define the amount of load to be applied to the model, thereby eliminating the convergence problems encountered in the static analysis. In addition, it can be used to simulate the deterioration and failure of materials, such as concrete cracking, in a better way. This type of analysis is direct integration of a dynamic process that uses the central-difference approach to march in pseudo-time [40]. By using explicitly central-difference time integration, the explicit dynamic procedures can efficiently perform a large number of small-time increments, eliminating the requirement for iterations and tangent stiffness matrices. The treatment of the contact problem is also simplified by conducting the proposed analysis. However, it should be mentioned that the inertial effects should be decreased when employing dynamic analysis for static problems by utilizing slow loading rates or by raising the mass density so that the oscillation of the findings is minimized [64].

### 3.7. Loading, Boundary Conditions, and Monitoring Points

The FE models used a loading technique and support steel plates comparable to those used in the experimental testing. The beams in the FE models were loaded using specified displacements located at the middle of the top surface of the steel loading plate, with a 0.1 mm increment. Line supports were placed at the center line of the bottom surface of the steel plates to prevent any relative movements in the vertical and transverse directions (z and y directions, respectively). The load was measured at the middle of the plate’s top surface, where the specified displacements were applied. Another point on the bottom surface of the concrete or composite structure at the mid-span of the beam was used to record the beam deflections. A strain monitoring point was used to measure the concrete compressive strains at the mid-span of the tested beams, similar to the location of the concrete strain gauges placed during the experimental tests. In contrast, strain in the tensile steel bars was monitored at a point placed at the mid-span of the RC beam specimen.

## 4. Results and Discussion

The experimental and numerical results pertaining to crack patterns, load-deflection behavior, and flexural strength are presented and discussed in this section. The FEMs were validated by comparing the numerical analysis results with the corresponding experimental test results.

### 4.1. Crack Patterns at Failure

#### 4.1.1. Uncorroded Un-Strengthened RC Beam (Control Specimen)

**Figure 8:**
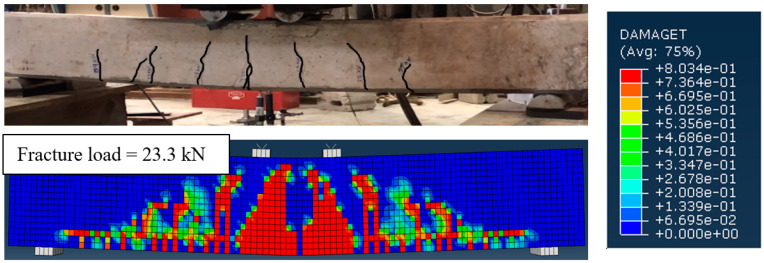
Experimental and numerical crack pattern for uncorroded control RC beam (UU).

Figure 8 shows the crack pattern at failure load predicted using FEM for the uncorroded un-strengthened RC beam (*UU*) compared to that obtained from the experimental test. It was observed that there is a high resemblance between the experimental and numerical crack patterns. Both experimental and numerical investigations indicated that the uncorroded control beam (*UU*) failed due to the yielding of the steel reinforcement bars, followed by concrete crushing.

#### 4.1.2. Corroded Un-Strengthened and Strengthened RC Beams

**Figure 9:**
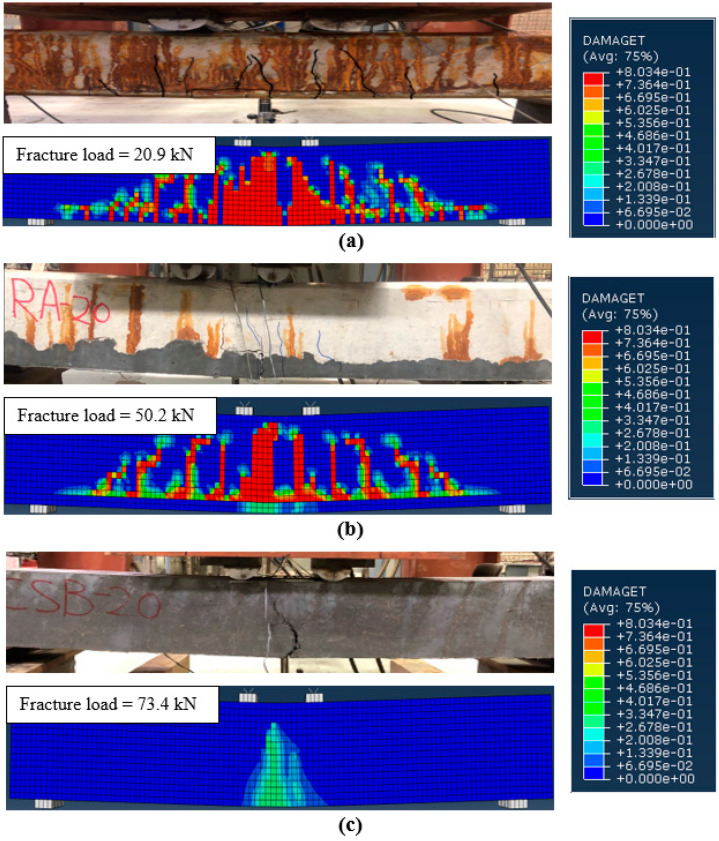
Typical experimental and numerical crack pattern results for (**a**) corroded un-strengthened beam (i.e., CUB); (**b**) 1-sided strengthened beam (i.e., CRA-20); and (**c**) 3-sided strengthened beam (i.e., CSB-20).

The crack patterns at failure load, predicted using the FEMs, were compared to that obtained from the experiments, as typically shown in Figure 9a–c for beams *CUB* (corroded un-strengthened), *CRA-20* (corroded one-sided strengthened), and *CSB-20* (corroded three-sided strengthened), respectively. It can be observed from Figure 9a that for the corroded un-strengthened beams, the experimental and numerical crack patterns closely matched together. Similar to the failure of the control RC beam (*UU*), both experimental and numerical results indicated that the corroded un-strengthened beams (*CUA*, *CUB*, and *CUC*) also failed due to steel yielding followed by concrete crushing. This finding implies that the damage of the main tensile steel bars due to reinforcement corrosion with actual mass loss of up to 21% did not change the ductile mode of failure of the RC beams. In addition, the flexural behavior of the UHPC-strengthened RC beams in the numerical simulation is similar to that observed during the experimental tests. Although only one major crack formed at the bottom and side surfaces of the UHPC layers in the three-sided strengthened RC beams due to the high tensile strength of the UHPC preventing any further cracks, several flexural cracks were observed in the one-sided strengthened RC beams. For the corroded strengthened beams also, a close matching of the crack patterns obtained from experimental tests and numerical modeling was observed, as shown in Figure 9b,c. This shows the effectiveness of the FEMs in predicting the flexural behavior of the corroded RC beam specimens, un-strengthened as well as strengthened. It can be concluded that the inclusion of the CDPM to model normal concrete and UHPC and the use of a cohesive surface bonding approach to simulate the interfacial bond stress-slip between the corroded bars and surrounding concrete enabled the FEMs to capture the flexural failure modes of the corroded strengthened RC beams appropriately.

### 4.2. Load—Deflection Behavior

#### 4.2.1. Uncorroded Un-Strengthened RC Beam (Control Specimen)

**Figure 10:**
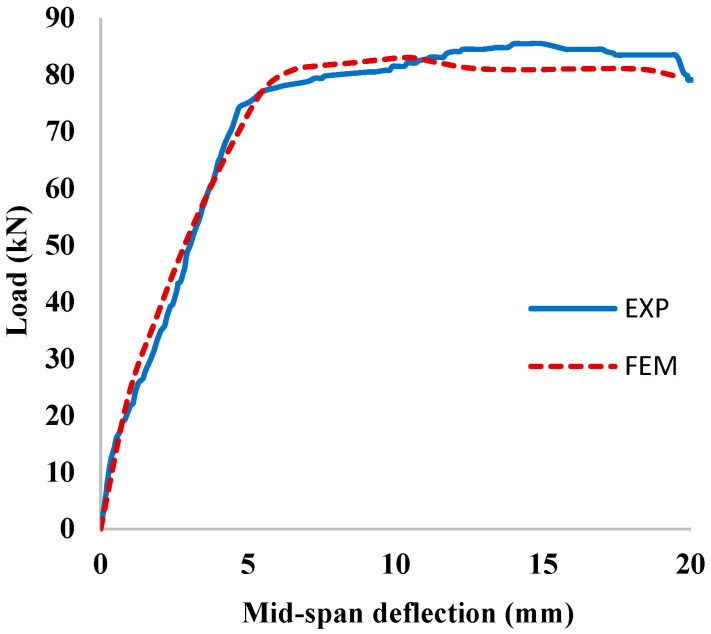
Comparison of the FEM and experimental results for uncorroded control beam (UU).

The load versus mid-span deflection curves of the un-corroded un-strengthened control RC beam (*UU*), obtained experimentally and numerically, are shown in Figure 10. It can be seen that the FEM captured the exact behavior of the uncorroded control beam (*UU*), including stiffness, yielding, cracking loads, and ultimate strength.

#### 4.2.2. Corroded Un-Strengthened RC Beams

**Figure 11:**
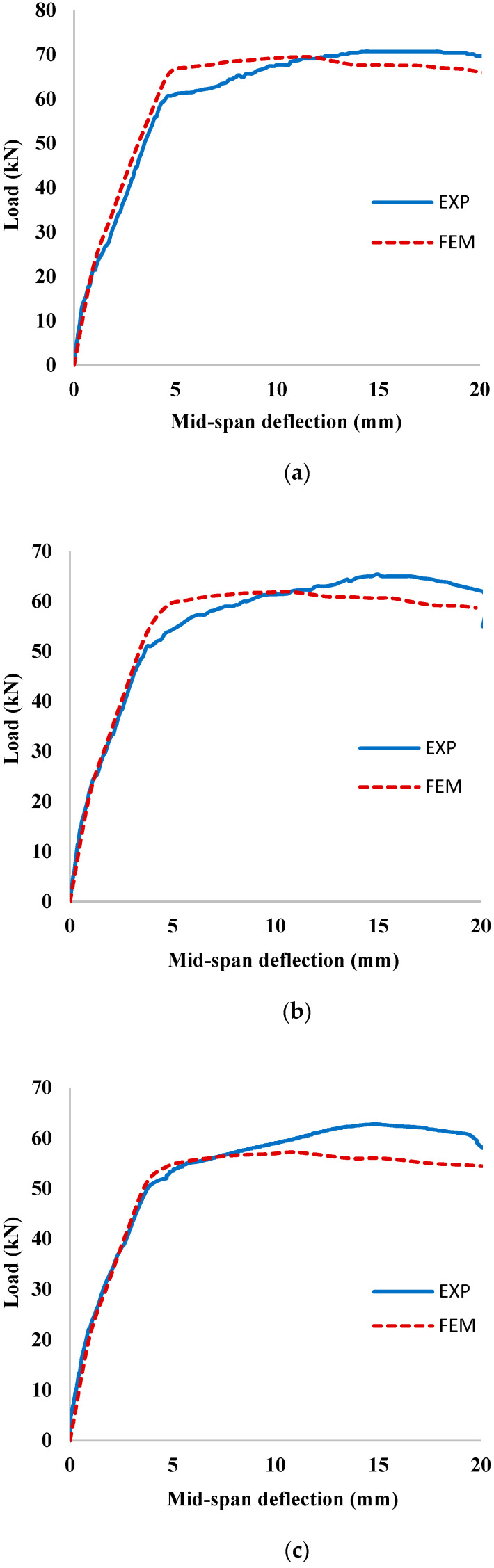
Comparison of the FEM and experimental results for corroded un-strengthened beams (**a**) CUA; (**b**) CUB; and (**c**) CUC.

The load versus mid-span deflection curves of the corroded un-strengthened RC beams obtained experimentally and numerically are shown in Figure 11a–c. It can be observed from Figure 11 that there is a good agreement between the experimental and numerical load-deflection curves, which confirms the accuracy of the FEMs in capturing the flexural behavior of the corroded RC beams. It confirms that the simulation of damage of the tensile steel bars due to reinforcement corrosion accounted in the FEM by considering a reduction in the yield strength and elastic modulus of the steel bars using a reduction factor is adequate for modeling the RC beams with corroded steel bars. The reason behind a slight deviation of the yielding point and slope of the softening part of the corroded un-strengthened RC beams may be attributed to the fact that the steel bars were modelled as elastic-perfectly plastic, whereas the behavior of steel bars was elastoplastic with small hardening as observed experimentally.

#### 4.2.3. Corroded Strengthened RC Beams

**Figure 12:**
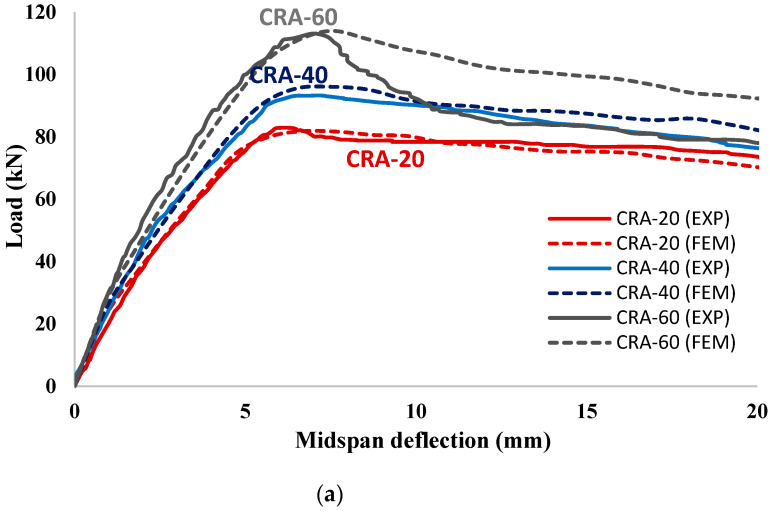

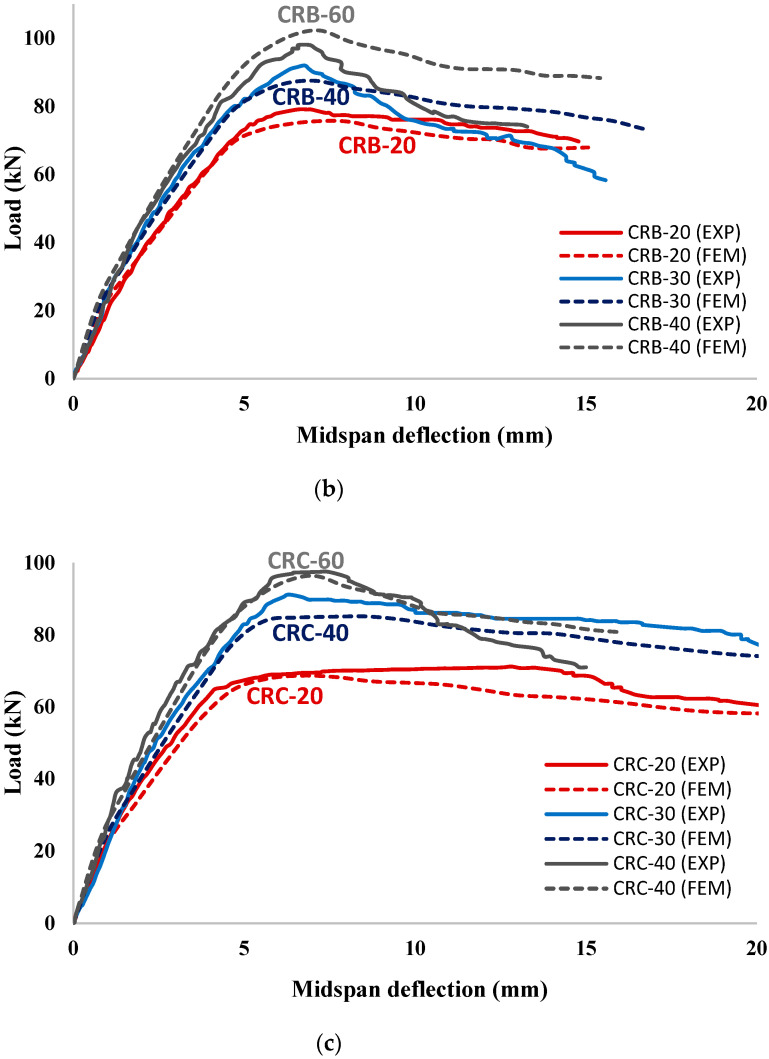
Comparison of the FEM and experimental results of 1-sided UHPC-strengthened RC beams for corrosion degree (**a**) group (A); (**b**) group (B); and (**c**) group (C).

**Figure 13:**
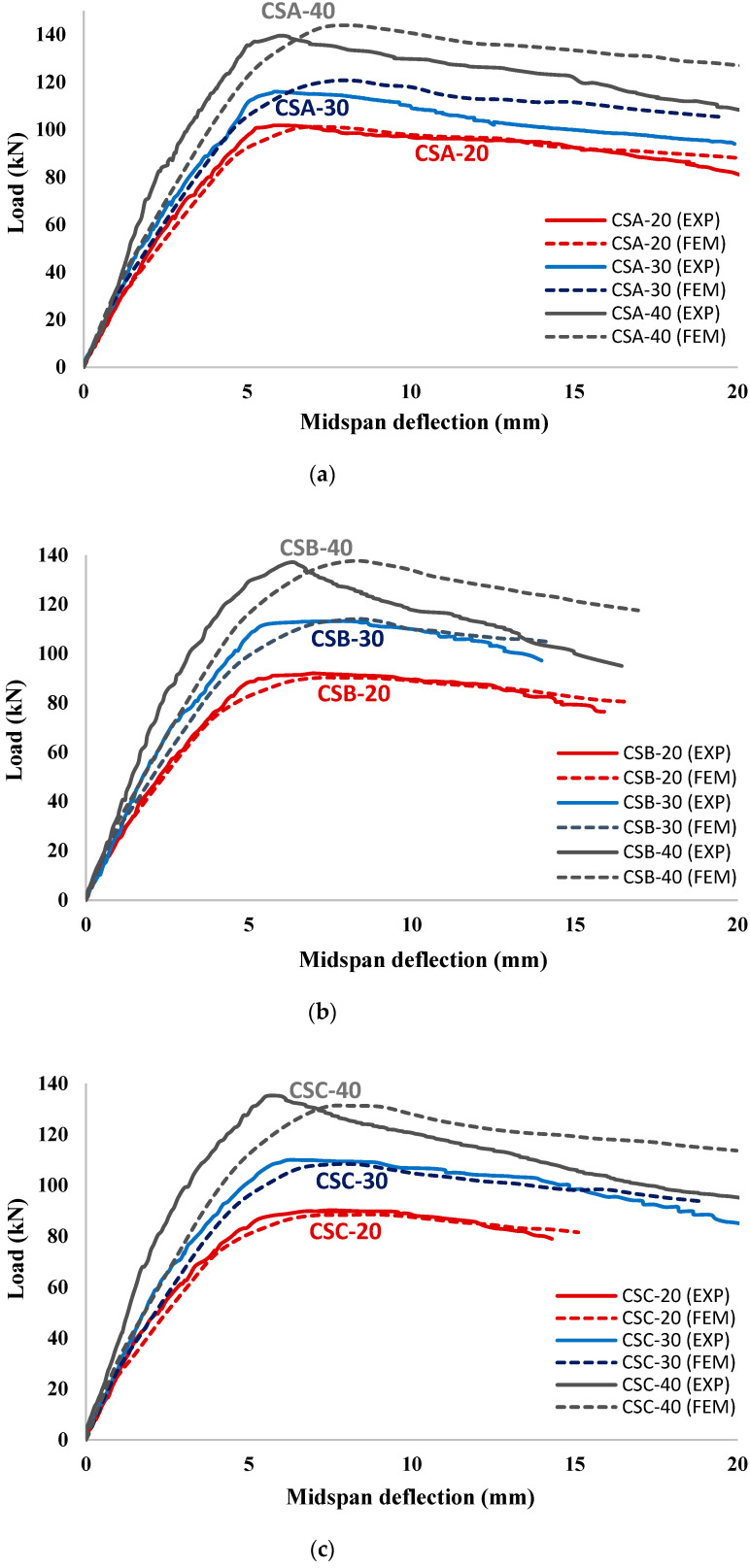
Comparison of the FEM and experimental results of 3-sided UHPC-strengthened RC beams for corrosion degree (**a**) group (A); (**b**) group (B); and (**c**) group (C).

The plots of load versus mid-span deflection curves of all strengthened RC beams obtained using the experimental and FEM are shown in Figure 12 (corroded one-sided strengthened RC beams) and Figure 13 (corroded three-sided strengthened RC beams) for comparison. It is noticed in Figure 12 and Figure 13 that the FEM can capture the flexural behavior (including the ultimate load-carrying capacity, ductility, and stiffness) of all corroded strengthened RC beams with a high degree of accuracy. It is to be noted that an excellent overall matching of the experimental and numerical results for the one-sided strengthened RC beams using the UHPC layer thicknesses of 20 and 40 mm, as shown in Figure 12. However, for the UHPC layer thickness of 60 mm the experimental and numerical results matched closely up to the ultimate load, thereafter, a small variation between experimental and FEM results softening part of the load-deflection curves. The discrepancy is most likely attributable to the use of a very high thickness of UHPC as a strengthening material from the bottom side of the beam specimens, with might cause a significant debonding at high loads [65]. Figure 13 shows the comparison of load versus mid-span deflection curves of the corroded three-sided strengthened RC beams obtained experimentally and numerically. Comparison confirmed an excellent overall matching of the experimental and numerical results for the strengthened RC beams using UHPC layers having thicknesses of 20 and 30 mm. However, similar to the case of one-sided strengthened RC beams, the three-sided strengthened RC beams using a higher UHPC thickness of 40 mm showed a small variation between the experimental and FEM results. The discrepancy at higher layer thickness may be attributed to the high thickness of UHPC that varies the concentration and orientation of steel fibers within the layers of UHPC [29]. This variation significantly affects the flexural behavior and stiffness of the strengthened RC beam [29]. However, the influence of concentration and steel fibers orientation is ignored in the FEM simulation.

### 4.3. Ultimate Load (Flexural Strength)

**Figure 14:**
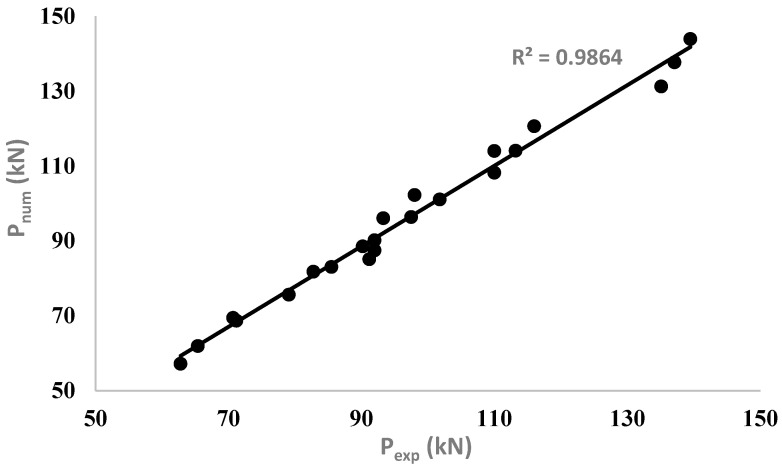
Comparison between experimental and numerical flexural strength results.

The ultimate loads obtained from FEMs are presented in Table 4 for comparison with the corresponding ultimate loads determined using the flexural testing. The comparison of the results indicates good agreement between the values of the ultimate load-carrying capacities for corroded un-strengthened, and strengthened RC beams in both configurations of UHPC layers, obtained experimentally and numerically. Figure 14 shows a strong fit between the experimental and numerical modelling values of the flexural strength results with a high *R^2^* value of 0.98, irrespective of the degree of corrosion and thickness and configuration of strengthening layers of UHPC. It confirms that the developed FEMs can capture the flexural behavior of the corroded strengthened and un-strengthened RC beams with a high degree of accuracy.

## 5. Parametric Study

The FEM analyses presented in this study demonstrate that the developed FEMs are capable of predicting the ultimate load-carrying capacities of the corroded strengthened RC beams with high accuracy. Therefore, the validated FEMs were used to carry out a parametric study to investigate the effect of varying the compressive strength of UHPC (used as strengthening material) and the thickness of UHPC layers on the flexural behavior of corroded strengthened RC beams.

### 5.1. Effect of Varying the Compressive Strength of UHPC

**Figure 15:**
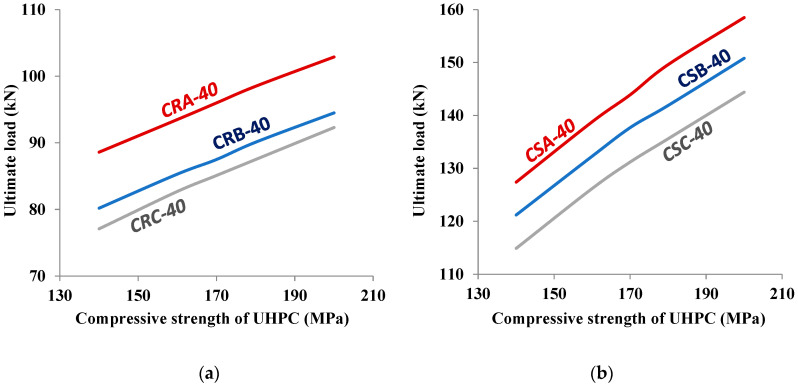
Variation of ultimate strength with compressive strength of UHPC strengthening at a typical layer thickness of 40 mm. (**a**) 1-sided, (**b**) 3-sided.

**Figure 16:**
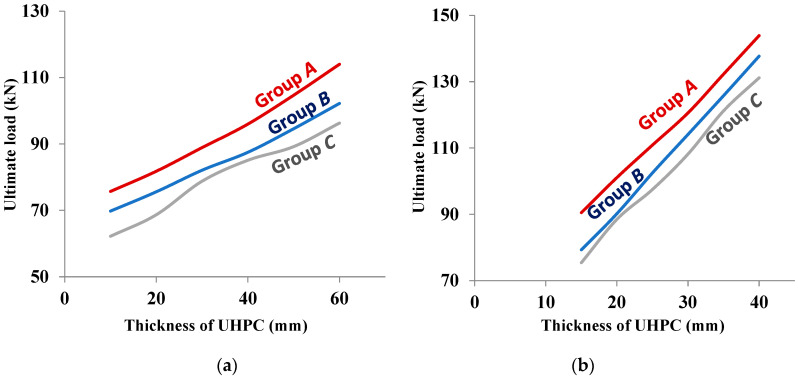
Variation of ultimate strength with thickness of UHPC strengthening layer at a typical compressive strength of 170 MPa. (**a**) 1-sided, (**b**) 3-sided.

The compressive strength of UHPC used for strengthening the corroded RC beams was 170 MPa. In order to examine the effect of varying the compressive strength of UHPC, two values of a compressive strength less than 170 MPa (i.e., 140 and 160 MPa) and two values of compressive strength more than 170 MPa (i.e., 180 and 200 MPa) were considered to predict the flexural behavior of corroded strengthened RC beams using FEMs. Figure 15a,b shows the plots of the predicted values of the flexural strength of corroded strengthened RC beams versus the compressive strength of UHPC used for both one-sided and three-sided strengthening of the beams, respectively, for different degrees of reinforcement corrosion at a typical layer thickness of 40 mm. It can be seen from the plots shown in Figure 15a,b that the compressive strength of UHPC has a significant effect on the flexural strength of the corroded strengthened RC beams. The flexural strength increased significantly with an increase in the compressive strength of UHPC. The steeply inclined curves shown in Figure 15b indicate that the beneficial effect of higher compressive strength of UHPC is more pronounced in the case of three-sided strengthening as compared to the one-sided strengthening for which the curves are flattened. Figure 16 also depicts the effect of the degree of reinforcement corrosion on the flexural strength of strengthened RC beams. As expected, the ultimate load-carrying capacity of the strengthened RC beams decreased with the increase in the degree of reinforcement corrosion, as indicated by highest flexural strength for CRA and CSA RC beams (corroded at lowest level) and lowest flexural strength for CRC and CSC RC beams (corroded at highest level).

### 5.2. Effect of Varying the Thickness of UHPC Layers

In order to examine the effect of the thickness of UHPC strengthening layers, the parametric study using the validated FEMs was carried out considering six different thicknesses of the UHPC layers: 10, 20, 30, 40, 50, and 60 mm for one-sided UHPC-strengthening configuration and 15, 20, 25, 30, 35, and 40 mm for three-sided UHPC-strengthening configuration. The compressive strength of the UHPC was typically taken as 170 MPa in the calculation of the flexural strength of the strengthened RC beams for all the considered layer thicknesses. Figure 16a,b shows the variation of flexural strength with layer thickness for one-sided and three-sided strengthened RC beams for all three degrees of reinforcement corrosion. The increase in the thickness of UHPC layers significantly increased the flexural strength of the corroded strengthened RC beams. The effects of the degree of reinforcement corrosion and configuration of UHPC strengthening layers on the flexural strength of strengthened RC beams were observed to be similar to that of the parametric study for the examination of the effect of compressive strength of UHPC. The beneficial effect of a higher layer thickness is more for three-sided strengthening, and the higher degree of reinforcement corrosion had a more adverse effect on the flexural strength. Figure 16 can be utilized to select the optimum thickness of the UHPC layer (with a compressive strength of 170 MPa) corresponding to the desired ultimate load-carrying capacity of the corroded RC beam for a given degree of reinforcement corrosion.

## 6. Conclusions

In this paper, the finite element modeling (FEM) using the concrete-damaged plasticity model (CDPM) was carried out to simulate the flexural behavior of corrosion-damaged RC beams strengthened with UHPC layers having different configurations and thicknesses. The numerical modeling results were calibrated and validated utilizing the experimental data developed in the present work by testing the corroded strengthened RC beams. The calibrated FEMs were used for parametric studies to examine the effects of varying the compressive strengths and thicknesses of the UHPC layers on the flexural behavior of corroded strengthened RC beams. The following conclusions were drawn based on the outcomes of the present work:

(1) The adoption of CDPM for simulating the normal concrete (used to cast the RC beams) and UHPC (for strengthening the corroded RC beams) and the selection of a cohesive surface bonding approach to simulate the bond between the corroded reinforcing bars and surrounding concrete were found to be appropriate in developing the FEMs, as evidenced by the high accuracy of the FEMs in simulating the flexural behavior of the corroded strengthened RC beams.
(2) The FEMs developed in the present study can capture the flexural behavior of the corroded RC beams strengthened using layers of UHPC with a high degree of accuracy, including crack pattern, ultimate strength, and failure mode. The accuracy of the developed FEMs was confirmed through the comparison of the results obtained experimentally with the corresponding results predicted using the FEMs.
(3) Parametric studies were carried out using the validated FEMs by varying the compressive strength and thickness of UHPC layers. The results of the parametric studies indicated that the flexural strength of corroded strengthened RC beams increases significantly with the increase in the compressive strength and thickness of UHPC layers and decreased significantly with the increase in the degree of reinforcement corrosion.
(4) The proposed FEMs can be utilized to select the optimum thickness of the UHPC layers to achieve the desired ultimate load-carrying capacity of the corroded RC beam for given degrees of reinforcement corrosion, UHPC layer configurations, and the compressive strength of UHPC.

## Figures and Tables

**Table 1:** Description of test specimens.

Specimen’s ID	Actual Mass Loss %	Strengthening Configuration	Thickness of UHPC Layer (mm)
*UU*	0	No strengthening	0
*CUA*	9.8		0
*CUB*	17.4	No strengthening	0
*CUC*	20.8		0
*CRA-20*	10		20
*CRA-40*	10.8	1-sided	40
*CRA-60*	11.8		60
*CRB-20*	18.1		20
*CRB-40*	17.8	1-sided	40
*CRB-60*	18.3		60
*CRC-20*	22.1		20
*CRC-40*	20.8	1-sided	40
*CRC-60*	22.6		60
*CSA-20*	10.2		20
*CSA-30*	9.5	3-sided	30
*CSA-40*	9.1		40
*CSB-20*	17.5		20
*CSB-30*	16.7	3-sided	30
*CSB-40*	16.8		40
*CSC-20*	21.2		20
*CSC-30*	22.3	3-sided	30
*CSC-40*	20.9		40

**Table 2:** Input parameter values for normal concrete and UHPC to define the CDPM.

Input Parameter	Normal Concrete	UHPC
Mass density (kg/m^3^)	2400	2500
Young modulus (GPa)	33	46
Poisson ratio	0.19	0.20
*Ψ* (°)	36	36
*ϵ*	0.1	0.1
$\frac{\sigma_{bo}}{\sigma_{co}}$	1.16	1.16
*K*	0.667	0.667
Parameter of viscosity	0	0

**Table 3:** Values of stiffnesses, $\tau_{max}$, and $S_{max}$ used in the FEMs.

Beam’s ID	$\tau_{max}$ (MPa)	$S_{max}$ (mm)	$k_{ss}\;$$=k_{tt}$ (N/mm^3^)	$k_{nn}$ (N/mm^3^)
*CRA-20*	8.95	0.396	22.58	2258.00
*CRA-40*	8.87	0.397	22.37	2236.82
*CRA-60*	8.78	0.397	22.10	2209.62
*CRB-20*	8.16	0.403	20.22	2021.86
*CRB-40*	8.19	0.403	20.31	2031.36
*CRB-60*	8.14	0.404	20.16	2015.51
*CRC-20*	7.77	0.411	18.91	1891.15
*CRC-40*	7.89	0.408	19.34	1934.39
*CRC-60*	7.72	0.412	18.74	1874.36
*CSA-20*	8.93	0.397	22.53	2252.75
*CSA-30*	9.00	0.396	22.71	2270.96
*CSA-40*	9.03	0.396	22.79	2279.23
*CSB-20*	8.22	0.403	20.41	2040.81
*CSB-30*	8.30	0.402	20.66	2065.76
*CSB-40*	8.29	0.402	20.63	2062.66
*CSC-20*	7.85	0.409	19.21	1921.16
*CSC-30*	7.75	0.411	18.84	1884.44
*CSC-40*	7.93	0.407	19.48	1947.56

**Table 4:** Ultimate load-carrying capacity of the RC beam specimens.

Beam ID	Experimentally	Numerically	Difference (%)
	$P_{exp}\;\left(kN\right)$	$P_{num}\;\left(kN\right)$	
*UU*	85.5	83.0	−2.9
*CUA*	70.7	69.5	−1.7
*CUB*	65.4	61.9	−5.4
*CUC*	62.8	57.2	−8.9
*CRA-20*	82.8	81.8	−1.2
*CRA-40*	93.3	96.0	2.9
*CRA-60*	110.0	114.0	3.6
*CRB-20*	79.1	75.6	−4.4
*CRB-40*	92.0	87.5	−4.9
*CRB-60*	98.0	102.2	4.3
*CRC-20*	71.2	68.7	−3.5
*CRC-40*	91.2	85.1	−6.7
*CRC-60*	97.5	96.4	−1.1
*CSA-20*	101.8	101.1	−0.7
*CSA-30*	116.0	120.6	4.0
*CSA-40*	139.5	143.9	3.2
*CSB-20*	92.0	90.2	−2.0
*CSB-30*	113.2	114.1	0.8
*CSB-40*	137.1	137.7	0.4
*CSC-20*	90.2	88.5	−1.9
*CSC-30*	110.0	108.2	−1.6
*CSC-40*	135.1	131.2	−2.9

## Data Availability

The data presented in this study are available upon the request from the corresponding author.
